# Simultaneous quantum yield measurements of carbon uptake and oxygen evolution in microalgal cultures

**DOI:** 10.1371/journal.pone.0199125

**Published:** 2018-06-19

**Authors:** Niu Du, Pardis Gholami, David I. Kline, Christopher L. DuPont, Andrew G. Dickson, Dominick Mendola, Todd Martz, Andrew E. Allen, B. Greg Mitchell

**Affiliations:** 1 Scripps Institution of Oceanography, University of California San Diego, La Jolla, California, United States of America; 2 J. Craig Venter Institute, La Jolla, California, United States of America; 3 Smithsonian Tropical Research Institute, Apartado, Republic of Panama; Mount Allison University, CANADA

## Abstract

The photosynthetic quantum yield (Φ), defined as carbon fixed or oxygen evolved per unit of light absorbed, is a fundamental but rarely determined biophysical parameter. A method to estimate Φ for both net carbon uptake and net oxygen evolution simultaneously can provide important insights into energy and mass fluxes. Here we present details for a novel system that allows quantification of carbon fluxes using pH oscillation and simultaneous oxygen fluxes by integration with a membrane inlet mass spectrometer. The pHOS system was validated using *Phaeodactylum tricornutum* cultured with continuous illumination of 110 μmole quanta m^-2^ s^-1^ at 25°C. Furthermore, simultaneous measurements of carbon and oxygen flux using the pHOS-MIMS and photon flux based on spectral absorption were carried out to explore the kinetics of Φ in *P*. *tricornutum* during its acclimation from low to high light (110 to 750 μmole quanta m^-2^ s^-1^). Comparing results at 0 and 24 hours, we observed strong decreases in cellular chlorophyll a (0.58 to 0.21 pg cell^-1^), Fv/Fm (0.71 to 0.59) and maximum Φ_CO2_ (0.019 to 0.004) and Φ_O2_ (0.028 to 0.007), confirming the transition toward high light acclimation. The Φ time-series indicated a non-synchronized acclimation response between carbon uptake and oxygen evolution, which has been previously inferred based on transcriptomic changes for a similar experimental design with the same diatom that lacked physiological data. The integrated pHOS-MIMS system can provide simultaneous carbon and oxygen measurements accurately, and at the time-resolution required to resolve high-resolution carbon and oxygen physiological dynamics.

## Introduction

Microalgae are capable of acclimating to dynamic light environments by reducing or increasing light harvesting capacity depending on light intensity, and implementing various strategies for stress mitigation when absorption of light exceeds photosynthetic carbon fixation capacity [1]. As a result, short-term photosynthesis vs. irradiance (P vs. E) responses are highly variable for different acclimation light conditions [2]. Several acclimation mechanisms at different levels of photosynthesis are involved in the physiological response to light, nutrient and temperature stresses. When exposed to super-saturating light that exceeds the photochemical capacity, acclimation mechanisms include, but are not limited to, non-photochemical quenching (NPQ) of excessively absorbed light energy as heat, and alternative electron transport, that redirects the fate of electrons for rebalancing NADPH/ATP ratios to satisfy other cellular energy requirements [3–7].

The quantum yield of photosynthesis (Φ), defined here as moles of inorganic carbon fixed or oxygen evolved per mole of photons absorbed, is an important physiological parameter that represents the photochemical efficiency of light utilization and is regulated by environmental factors that control growth and acclimation. In general, variable chlorophyll fluorescence photon flux relative to the maximum yield under actinic light ($\Delta \phi_{F}/\phi_{F_{m}}$) has been accepted as a good estimation of Φ, since a linear relation was discovered between them [3]. Note that the symbol Φ refers to the mass of carbon or oxygen per unit of absorbed photons whereas ϕ is a relative fluorescence flux. The term $\Delta \phi_{F}/\phi_{F_{m}}$ is mathematically equivalent to the product of photochemical fluorescence quenching and the efficiency of excitation capture by open PS II reaction centers (F_v_/F_m_). Both $\Delta \phi_{F}/\phi_{F_{m}}$ and F_v_/F_m_ are widely used because fluorescence measurements are easy, fast, non-destructive, provide biophysical information, and inference about biochemical information, at time scales of seconds or faster which is extremely useful for some research questions. However, fluorescence flux is not directly an indicator of mass flux, and therefore it does not represent the full potential of photosynthesis, especially for the dark reactions [4]. Furthermore, differences in variable fluorescence protocols can give different values of $\Delta \phi_{F}/\phi_{F_{m}}$ and Fv/Fm for the same culture [5]. In contrast, mass exchange measurements can provide quantitative estimates for computing the actual mass fluxes relative to quanta absorbed, however, a larger quantity of biomass is required for these types of methods, their implementation is much more difficult to achieve and they are not able to resolve photosynthetic processes at time-scales of seconds.

In algal physiology studies under a dynamic environment, a mass flux-based approach is necessary to be able to explore changes in Φ. For example, Broddrick et al. [6] used oxygen flux as a constraint in a cyanobacterium genome scale model (GEM) to simulate whole cell metabolic flux, which enabled the discovery of unique light-driven cellular mechanisms. While oxygen evolution was directly measured in that study, carbon uptake was estimated simply in direct stoichiometric proportion to the oxygen evolution. Since previous studies on photosynthetic quotients (PQ, the ratio of oxygen evolved to carbon fixed) have shown that this ratio can be highly variable, depending on environmental factors that regulate physiology and growth, and algal species. [7–10]. Therefore, deviations in the ratio of oxygen evolution and carbon uptake would not have been resolved by the metabolic model of Broddrick [11].

Despite the well-recognized need for simultaneous and quantitative estimates of carbon and oxygen fluxes to develop a deeper understanding of energy and mass flux in both photosynthesis and respiration, previous studies have not yet established robust methodologies that are capable of generating fast, quantitative and high precision results for both carbon and oxygen [11,12] (details discussed in S1 Text). Here we describe a novel integrated hardware and software system to monitor carbon and oxygen dynamics at short time scales (minutes) using pH oscillation (pHOS) that is validated relative to an independent estimate of net carbon growth of a well-studied marine diatom *Phaeodactylum tricornutum* (*P*. *tricornutum*) [13]. The pHOS system was integrated to a membrane inlet mass spectrometer (MIMS) and we used the integrated pHOS-MIMS system for a 24 hour acclimation study in which the cultivation light was shifted from sub-saturating to super-saturating at time 0 to resolve the kinetics of Φ for both net carbon and net oxygen during the acclimation. The results were interpreted in the context of a similar study using the same organism that reported the changes in gene expression over 24 hours determined by transcriptomic analysis [14].

### Principles of DIC measurement dynamics using pHOS

The dissolved inorganic carbon (DIC) pool in an aqueous medium includes three carbon species (CO_2_, HCO_3_^-^ and CO_3_^2-^) whose relative concentrations change significantly between pH 6–10. For a closed microalgal culture system, changes in DIC reflect the dynamics of carbon transport into and out of the algal cells. Early work by Allen and Spence[15] measured carbon uptake by submerging freshwater plants and microalgae into NaHCO_3_ solutions and tracking the pH change over time. Their study was the first to demonstrate the feasibility of using “pH drift” caused by changes in carbonate chemistry as a method for photosynthetic carbon uptake measurement in aqueous media. During photosynthesis, DIC from the aqueous medium enters the algal cells either via passive diffusion of CO_2_ or active transport of HCO_3_^-^. CO_2_ is then fixed into organic molecules via the Calvin-Benson cycle. The assimilation of CO_2_ (Eq 1), and subsequent formation of OH^-^ ions from the carbonate system re-equilibration (Eq 2), together result in a net pH increase in the medium.

$$CO_{2}+H_{2}O\to Organic\;carbon+O_{2}$$ (1)

$$CO_{2}+OH^{-}⇐HCO_{3}^{-}\Leftrightarrow CO_{3}^{2-}+H^{+}$$ (2)

However, the change in pH does not track the DIC concentration in a simple linear relationship. To estimate carbon uptake, Allen and Spence [15] assumed the total alkalinity (A_T_) to be a constant value, and calculated DIC using Eq 3:

$$DIC=\frac{A_{T}-[OH^{-}]+[H^{+}]}{\alpha_{1}+2\alpha_{2}}$$ (3)

Where α_1_ and α_2_ are the ionization fractions of HCO_3_^-^ and CO_3_^2-^ in fresh water medium, respectively.

Carbonate chemistry in seawater-based culture media is far more complex than the fresh water based system that Allen and Spence [15] worked with, due to higher salinity and more complex ion composition. Relatively recent advances in understanding the complex carbonate chemistry in seawater [16], combined with advances in pH sensors and electronic circuits, have made possible the application of a “pH drift” system for microalgae in sea water media. After reviewing prior experimental assumptions and conditions, we modified the original “pH drift” concept to a pH oscillation method, adding the following improvements over those employed by Allen and Spence [15]:

1. We shortened the measurement time from a few hours to 30 minutes to strengthen the assumption of a constant A_T_, given that algal cell growth within the time scale of hours can change A_T_, especially for high cell density lab cultures [17,18];
2. Using a high sensitivity pH apparatus and oscillating light/dark periods of relatively short duration (2 min), we were able to control the pH using the oscillation method over a very small range (usually less than 0.1 pH units), thus avoiding significant changes in media carbonate chemistry that affects the DIC uptake kinetics; and (3) For measurements in the pHOS-MIMS system we gently centrifuged the cells into a pellet, and re-suspended them in the fresh culture media for which careful calibrations had been carried out in order to minimize changes in chemistry that might affect the cell physiology and for precise understanding of the carbonate chemistry and alkalinity.

An example of pH oscillation during a series of measurements over a 30 minute period (1800 s) is demonstrated in S1 Fig. The calculations for carbon speciation were based on the following equations from Dickson et al. [19]:

$$DIC=[HCO_{3}^{-}]+[CO_{3}^{2-}]+[CO_{2}]$$ (4)

$$[CO_{2}]=\frac{A_{C}{\left[H^{+}\right]}^{2}}{K_{1}([H^{+}]+2K_{2})}$$ (5)

$$[HCO_{3}^{-}]=\frac{A_{C}[H^{+}]}{[H^{+}]+2K_{2}}$$ (6)

$$[CO_{3}^{2-}]=\frac{A_{C}K_{2}}{[H^{+}]+2K_{2}}$$ (7)

Where A_c_ is the carbonate alkalinity, and K_1_ and K_2_ are the dissociation constants of HCO_3_^-^ and CO_3_^2-^ in aquatic solutions as functions of salinity and temperature, respectively (details in S2 Text). A_c_ is related to A_T_ and varies as a function of pH.

## Materials and methods

### Cell cultivation and sampling

The marine diatom *P*. *tricornutum* CCAP 1055/1 (purchased from The Culture Collection of Algae and Protozoa) was cultured at 25°C with Artificial Sea Water medium (ASW; http://www3.botany.ubc.ca/cccm/NEPCC/esaw.html), under continuous white LED light (24h day^-1^) at an intensity of 110 ± 10 μmole quanta m^-2^ s^-1^. The cells were collected during mid-log exponential growth phase, then washed and re-suspended in fresh ASW medium to make up a series of differing cell concentration solutions. From the same culture we also determined the growth rate, particulate organic carbon and Chla and determined the rate of Chla specific carbon growth for a 5 hour period for 6 separate cultures. OD_750_ was measured each hour during this to determine the growth rate. This data provided conversion coefficients for POC (175 ± 21 μg OD_750_^-1^ cm^-1^), and Chla (8.31 ± 0.73 μg OD_750_^-1^ cm^-1^), which were used in Eq 9 as part of the validation of the pHOS estimates for carbon fixation. For the low-to-highlight acclimation experiment, the cells were cultured at continuous 110 μmole quanta m^-2^ s^-1^ LED light for more than 6 division cycles (~3 days) then shifted to 750 μmole quanta m^-2^ s^-1^ LED light for 24 hours. During the high light exposure samples were taken at time 0, 1, 3, 6, 12 and 24 hours for simultaneous carbon uptake and oxygen evolution measurement with the pHOS-MIMS system, and spectral absorption.

### P vs. E

Cells harvested by centrifugation were re-suspended in ASW medium with known alkalinity were inoculated into the glass cell of an ALG instruments (http://manualzz.com/doc/7032749/instrument-manual) with the integrated pH oscillation (pHOS) and membrane inlet mass-spectrometer (MIMS) system for simultaneous pH and dissolved oxygen and argon measurements. The mixing, light and temperature control for the measurement was achieved by a ALG instruments that has been validated previously [20,21]. During measurement, samples were treated with alternative dark/light periods with 2 min interval and at increasing light intensity steps of 0, 20, 50, 200, 500, 1000, and 2000 μmole quanta m^-2^ s^-1^. Measurement of pH, oxygen and argon were recorded at 1 Hz during the dark/light periods for computing DIC and oxygen concentrations and subsequently the rates of change. Rates of both parameters measured during the first dark period (light intensity = 0 μmole m^-2^ s^-1^) and the following light periods were used to create the P vs. E curves. For the curve fitting we offset the respiration rate to force the data to start at 0 so we could use the Platt et al. [22] function that cannot use negative values. Then the fitted result was subtracted by the offset respiration value, following Richardson et al. [23].

### Chlorophyll a-specific absorption coefficient

*In vivo* whole cell absorption was determined at 1 nm wavelength (λ) intervals using a dual beam spectrophotometer (Cary 100) equipped with a 30 cm Lab Sphere integrating sphere [24]. The sample was placed in a 1 cm path length cuvette. The chlorophyll a-specific absorption coefficient a*_ph_ (λ) was estimated using Eq 8.

$$a_{ph}^{*}\left(\lambda \right)=\frac{\ln\left(10^{A(\lambda )}\right)}{[Chla]x}$$ (8)

where A(λ) is the spectral absorbance measured with the spectrophotometer integrating sphere, and x is the path length of light absorption. Chlorophyll a concentration was measured with a calibrated Turner 10-AU fluorometer after all pigments were extracted in 90% acetone.

### Quantum yield (Φ) calculation

Φ was calculated for both carbon uptake and oxygen evolution using the definition of net photosynthsis, modified from Sosik and Mitchell [25] (Eq 9).

$$\phi =\frac{P_{Chl}}{\int_{400nm}^{700nm}a_{ph}^{*}(\lambda )E_{0}(\lambda )d\lambda }$$ (9)

Where P_chl_ is the net photosynthesis per unit of Chla (we did not resolve gross photosynthesis or respiration separately), and the integrated product of a*_ph_ (λ) and spectral irradiance E_0_ (λ) is the total photon flux absorbed.

### Particulate carbon and nitrogen (POC/PON)

For POC and PON estimates, a 10 ml sample was filtered onto a 0.2 μm Nuclepore polycarbonate filter under vacuum pressure < 5 PSI, and the concentrated biomass was then washed into a 25 ml acid-washed TOC glass vials with 20 ml DI water. A blank was determined by filtering an equal volume of DI water and treating the sample the same. The suspended samples and blanks were then analyzed with a Shimadzu TOC-L Analyzer using the total carbon and nitrogen (TC/TN) protocol and the POC and PON determined after subtracting the value for the blank.

### pHOS system

**Fig 1:**
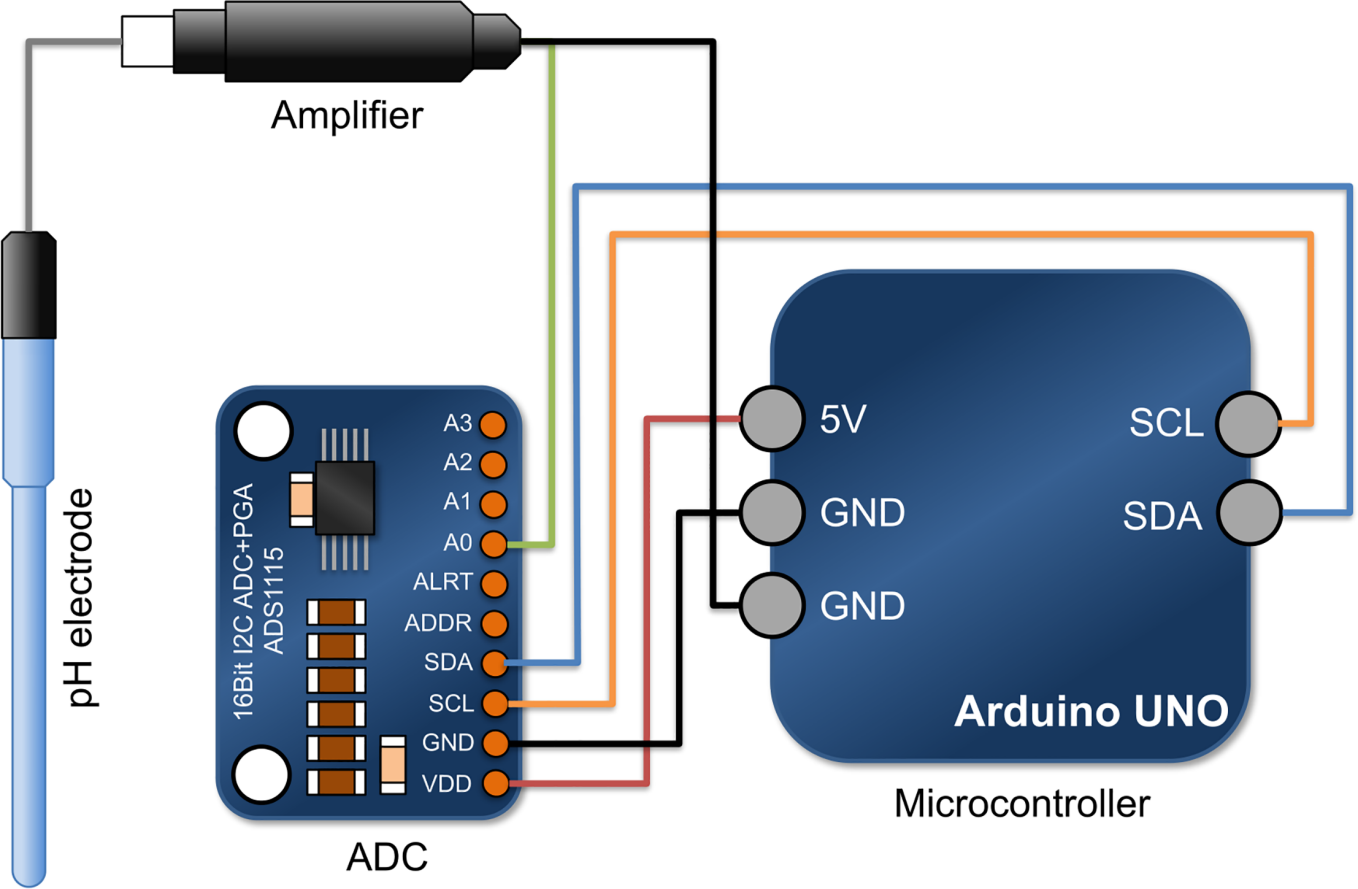
The pHOS apparatus for pH calibration, measurement and data logging. The configuration of nodes on the Arduino UNO controller was simplified to highlight the circuit connections. To improve the precision of pH measurement, the Arduino micro-controller must be plugged in with an independent power supply to avoid reference voltage fluctuation when powered only by the USB connection.

pH measurement was achieved using a Cole-Parmer glass liquid-filled pH electrode with internal reference (EW-05991-61). An electrode amplifier (Vernier model EA-BTA) was connected in-line to amplify the signal at 2.2V V^-1^ ratio within an input range of -450 to +1100mV (DC), with +1200mV DC offset on the output. The analog voltage signal was converted to a digital output using a 16 bit, analog-to-digital converter (ADC; ADA Fruit model ADA-1115), which in-turn, was connected to an Arduino UNO board via an integrated I^2^C circuit bus (Fig 1). The 16bit ADC has logic detection between 2.0–5.5 V (DC) and enables 0.1875mV/bit resolution at a default of “two-thirds” gain setting, allowing the system to measure pH changes of ±0.0015 pH unit resolution. A modified Arduino IO library (MathWorks, MA, USA) was uploaded to the Arduino controller to allow interactive communication via a USB cable between the Arduino circuit board and MATLAB based GUI application programmed on a laptop computer. In addition the Arduino UNO board has a 1 Amp 12V (DC) regulated power supply that was used to apply an extremely stable 5V signal as a reference for the pH voltage. All electronic parts, except for the pH electrode, were enclosed in a stainless steel box to provide electro-magnetic shielding.

### pHOS-MIMS integration

**Fig 2:**
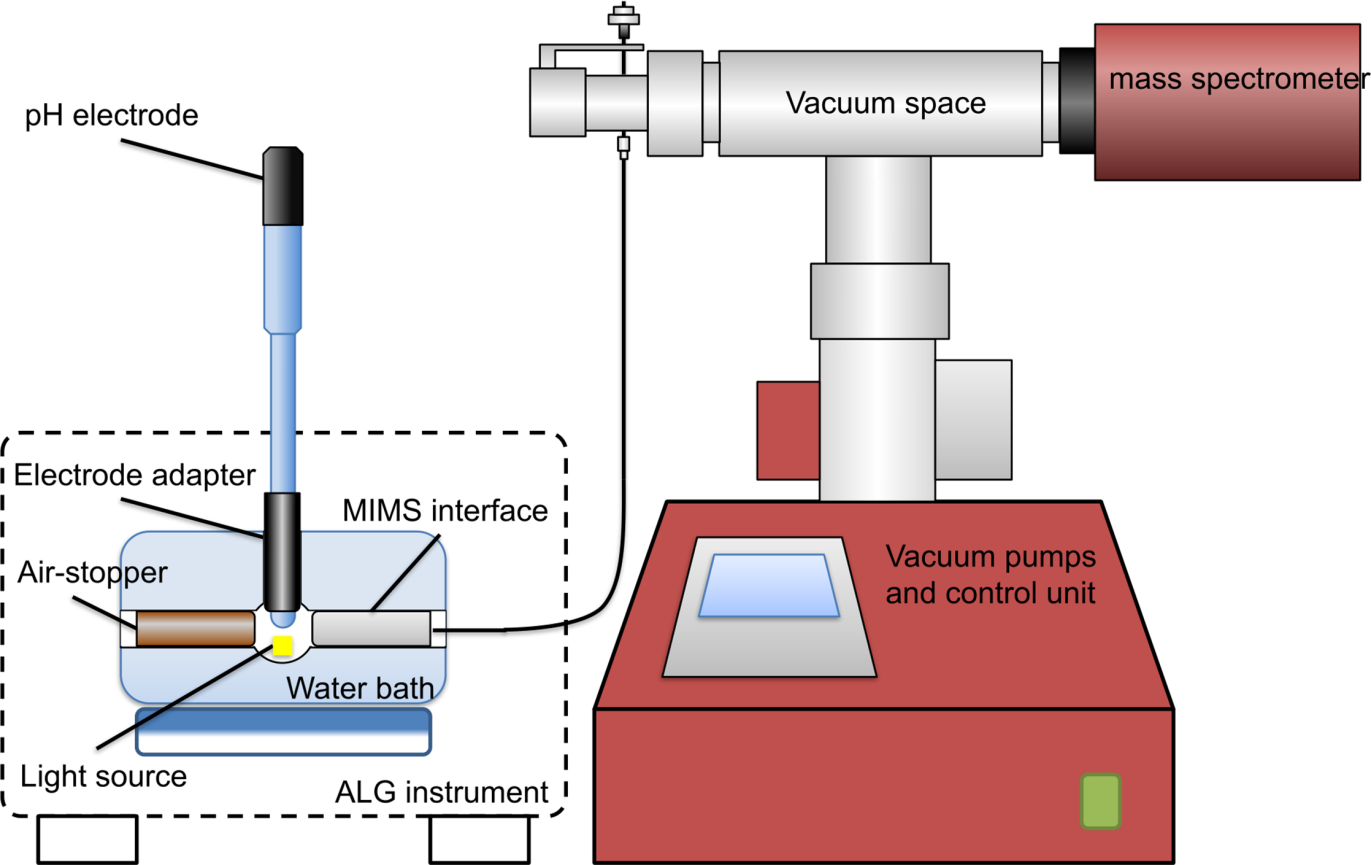
Integration of pHOS and MIMS system with the ALG instruments. The dashed line shows the size and shape of an ALG instruments bioreactor control unit in the background.

For simultaneous pH oscillation and oxygen production and consumption estimates, the pHOS system was integrated with a custom-assembled Membrane Inlet Mass-Spectrometry (MIMS). For interfacing, we used an ALG instruments bioreactor for light and temperature control. The ALG instruments bioreactor vessel is approximately 1 cm interior diameter and has three ports for normal operations, two on the sides for dual Clark-type electrodes and a port on the top for filling / removing the experimental sample. We modified our unit to include a pH electrode inserted into custom fabricated 3-D printed Plexiglas adapter that also served as the seal for the top standard taper opening. We also fabricated a custom probe to interface between one of the side ports of the sample cell and the vacuum space of the MIMS spectrometer. The proximal end of the MIMS probe head was covered with a 100-μm thick polydimethylsiloxane (PDMS) membrane sealed onto the head with a neoprene O-ring, which also served to seal the port. The port on the opposite side of the MIMS port was sealed by a rod with an O-ring but no probe (Fig 2). The MIMS allowed us to simultaneously measure oxygen and argon in the media solution. The inert gas argon was used as a reference to adjust the oxygen signal that is very sensitive to small, high frequency fluctuations in the vacuum pressure of the mass spectrometer [26]. We chose not to use the standard Clark-type electrode provided by the manufacturer for the ALG instruments because precipitation onto the electrode can result in greater resistance and a current that has a small interference with the pH measurements. While this can be managed by keeping the oxygen electrode well-polished, the MIMS provided very sensitive, accurate and stable oxygen estimates with no interference to the pH electrode and in the future we plan to use MIMS for other gases. Arduino driver and the computer programs for pHOS are available at https://github.com/ndu-UCSD/pHOS.

### pH electrode calibration

To ensure reliable pH measurements, the pH electrode’s response voltage for different pH standards was tested against predicted Nernst equation values (S3 Text). After the electrode response testing, a voltage to pH response was calibrated immediately preceding each pH measurement. The calibration procedure consists of two steps: first, a 3-point standard pH calibration with standard laboratory pH buffers, and second, conversion of each pH measurement from the National Bureau of Standards (NBS) scale to the Total scale based on a single measurement in Tris-ASW. The second step was necessary because of the liquid junction potential (LJP), which causes the measured [H^+^] to deviate from its true value due to changes in ionic strength and H^+^ activity, as the probe is moved from buffer solution to the ASW medium. The deviation of pH results from changes in E_0_ in the Nernst equation [27]. Considering that the chemical composition of the ASW medium is similar to the Tris-buffered synthetic seawater we used (Dickson lab, SIO), we assumed equal E_0_ between these two media. The Total scale pH (pH_T_) can then be calculated from measured pH (pH_m_) using Eq 10.

$$pH_{T}=pH_{m}-(pH_{m-Tris}-pH_{cal-Tris})$$ (10)

Based on the definition of pH_T_ described in Dickson [27], we assigned pH_T_ to be the “true” pH that reflected the [H^+^] contributed in a carbon speciation calculation, with an offset determined from the measured Tris buffer pH (pH_m-Tris_), and a calculated Tris buffer pH (pH_cal-Tris_). The pH of the Tris-buffered synthetic seawater was calculated following DelValls & Dickson [28] as a function of salinity (S) and temperature (T) in Kelvin units, using Eq 11.

$$\begin{array}{l} \mathrm{pHTris}=(11911.08‑18.2499S‑0.039336S2)/T\\ \;+(‑366.27059+0.53993607S+0.00016329S2)\\ \;+(64.52243‑0.084041S)\ln(T)‑0.11149858T \end{array}$$ (11)

### Alkalinity measurements and calibration

The total alkalinity (A_T_) in ASW medium is due to a variety of different chemical components but dominated by carbonate, phosphate and borate species. We followed the definition of A_T_ in Dickson et al. [16] and simplified the equations to represent only the relevant components in ASW medium (Eq 12).

$$\begin{array}{l} A_{T}=[HCO_{3}^{-}]+2[CO_{3}^{2-}]+[B{\left(OH\right)}_{4}^{-}]+[HPO_{4}^{2-}]+2[PO_{4}^{3-}]+[OH^{-}]\\ \;-[H_{3}PO_{4}]-{\left[H^{+}\right]}_{f}-[HSO_{4}^{-}] \end{array}$$ (12)

Additionally, removal of CO_2_ from the ASW medium can cause changes in chemical concentrations within the medium, however, A_T_ remains constant regardless of the CO_2_ budget, as a result of mass and charge balance within the system [16]. The measured [H^+^] in Total scale includes both free hydrogen ion [H^+^]_f_ and [HSO_4_^-^] [28]. Following the definition of carbonate alkalinity A_c_ in Eq 13 [19], we calculated the instantaneous A_c_ using Eq 14 by combining Eq 12 and Eq 13:

$$A_{C}=[HCO_{3}^{-}]+2[CO_{3}^{2-}]$$ (13)

$$A_{C}=A_{T}-[B{\left(OH\right)}_{4}^{-}]-[OH^{-}]-[HPO_{4}^{2-}]-2[PO_{4}^{3-}]-[SiO{\left(OH\right)}_{3}^{-}]+[H_{3}PO_{4}]$$ (14)

A_T_ was measured using the Gran titration method [29], with HCl standardized against reference seawater of known A_T_ (Dickson Lab, SIO). Alkalinity measurements were conducted before and after the measurement for verifying the assumption of constant A_T_ (Table 1). Since 1 mL from the bioreactor is not sufficient to titrate for A_T_, a parallel sample of medium with equivalent cell density was placed inside a clear 125ml Nalgene^®^ polyethylene bottle and treated with the same light source used for measurement of the P vs. E culture. Then 50 mL was taken from this reference to enable sufficient volume for titration to determine A_T_ post- incubation.

**Table 1:** Alkalinity calibration constants and results.

HCl (n = 3)	Medium density	Reference A_T_	Tris buffer pH	Medium A_T_ (n = 3)	A_T_ after 30min (n = 3)
0.1723 ± 0.0011 mol L^-1^	1.0352 kg L^-1^	2216.9 μmol kg^-1^	8.0936	1980.1 ± 7.4 μmol kg^-1^	1979.1 ± 8.8 μmol kg^-1^

Other alkalinity contributors were calculated using knowledge of the pH and the total molar concentrations of the elements [16]. Based on titration results during methods development, we determined that biological activities for boron and phosphorus species would have no significant effects on their respective concentrations during the 30-minute measurements. Therefore the total concentration for each of these two species was considered to be constant in the alkalinity calculations (Table 1).

### Detection limit

**Fig 3:**
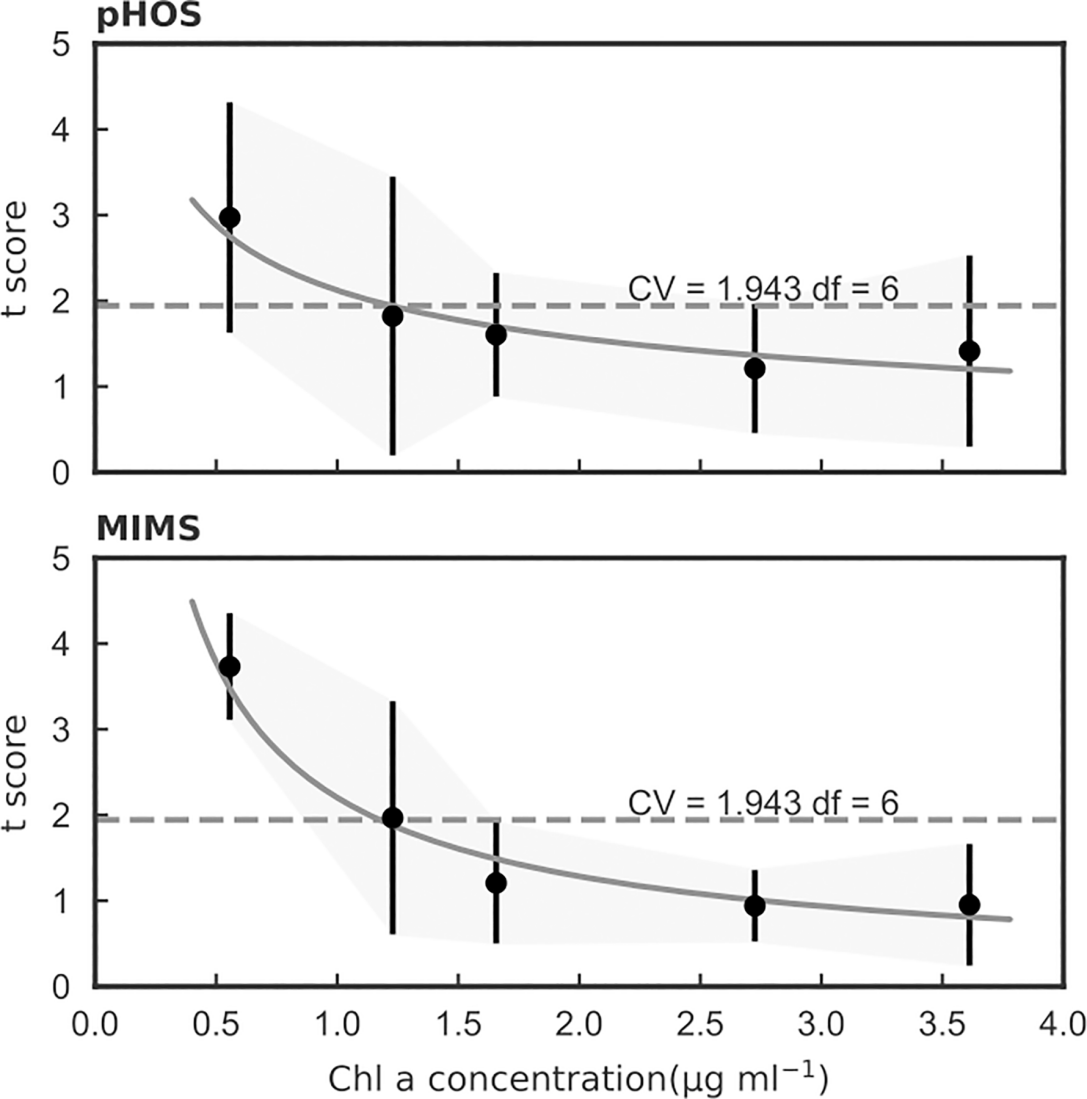
t-scores of pHOS and MIMS measurements as a function of Chla concentration. The critical value (CV = 1.943) for 95% confidence in replicates is marked with a dashed line. For Chla > 1.5 μg mL^-1^ replicated measurements are not significantly different.

Algal cell densities used in laboratory cultures are very high relative to natural aquatic systems. These high cell densities can result in significant light attenuation during the measurements, affecting photosynthesis-irradiance responses. In order to determine the sensitivity of our carbon uptake measurements to Chla concentration and light intensity, we tested 5 different Chla concentrations at 7 different light intensities (S2 Fig). A t-score was calculated for each Chla concentration, and the corresponding critical value (CV) from the standard t-table suggests that a t-score value less than 1.943 corresponded to a Chla concentration >1.5 μg mL^-1^ that is sufficient to provide statistically significant results (Fig 3).

**Fig 4:**
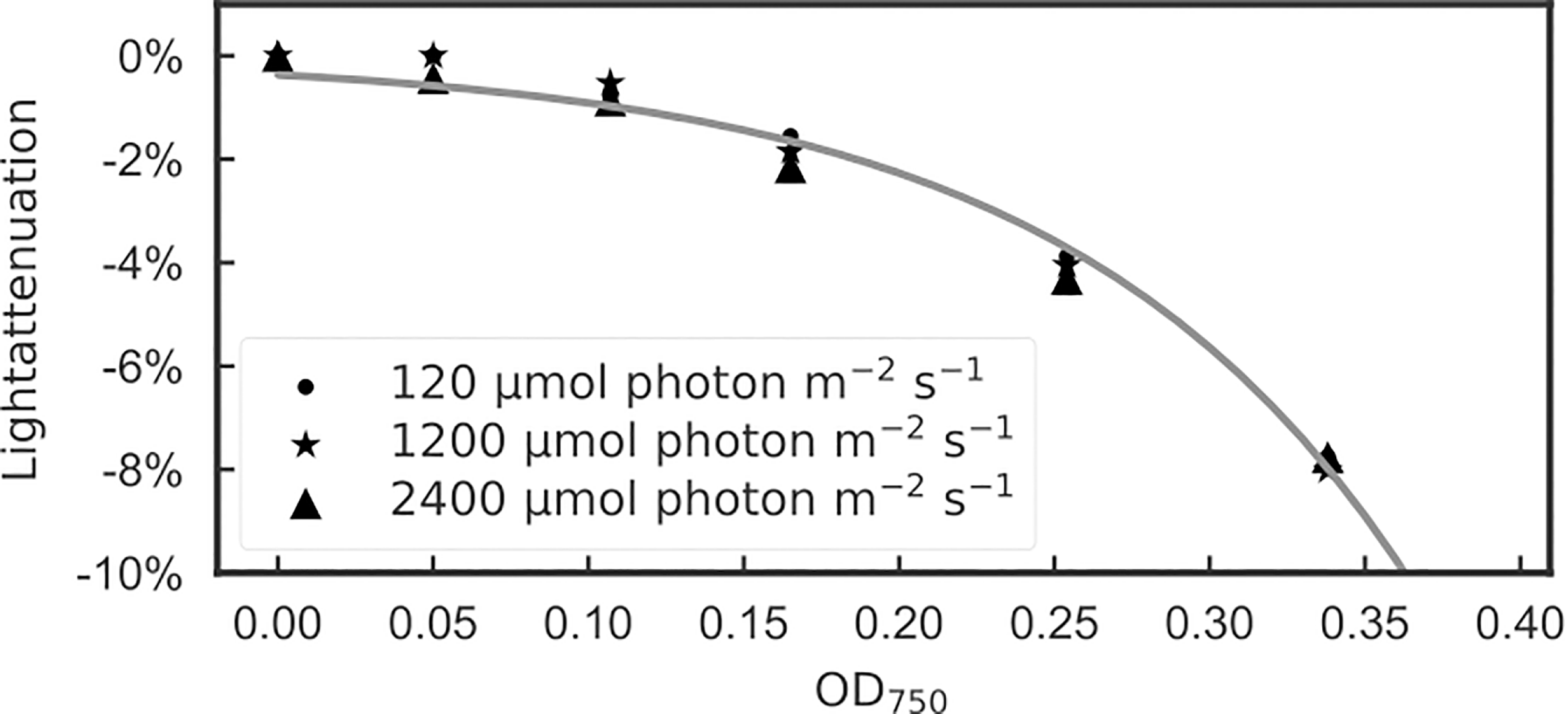
Empirical determination of PAR light attenuation within the ALG instruments measurement cuvette as a function of OD_750_. For OD_750_ less than 0.35 the effect of culture density has less than 10% reduction of PAR.

The Chla specific oxygen evolution and carbon uptake vs. irradiance responses suggest that the minimal Chla concentration should be greater than 1.5 μg ml^-1^ for this method to be effective (Fig 3). To account for possible errors in light intensity in the measurement vessel, we measured light attenuation for these samples at the center of the ALG instruments sample cuvette using a Li-Cor 250A photosynthetically active radiation (PAR) meter equipped with a WALZ 4π sensor. We then fit the light attenuation (E_attenuation_) as a function of OD_750_ (Fig 4; Eq 15):

$$E_{attenuation}=-e^{a\times OD_{750}-b}$$ (15)

Where *a* and *b* are arbitrary coefficients of the power function for attenuation in the cuvette scaled by a simple observation of OD_750_. Using a least squares fit we calculated values of a = 9.12 and b = 5.61 with r^2^ = 0.9839. For physiological measurements we limited light attenuation to no more than 10% of the total PAR measured so that it would have minimal impact on the results. This maximum allowed attenuation of PAR of 10% corresponded to OD_750_ = 0.35 cm^-1^. While an OD_750_ of 0.35 cm^-1^ would attenuate > 10% of PAR in a simple collimated 1 cm path, the ALG instruments bioreactor illumination had a complex optical geometry with several reflective surfaces (the sample vessel and the water jacket) such that reflected light re-entered the sample. We carried out a careful empirical calibration using the PAR meter inside the sample space measuring different positions and for different cell densities of *P*. *tricornutum* to derive coefficients in Eq 15 and to define the upper limit of OD_750_ to ensure minimal PAR attenuation.

### Oxygen response calibration

The response to changes in oxygen concentration was calibrated by establishing saturation and zero oxygen levels. The oxygen concentration was then estimated from a linear fit between these two points. The medium used for the saturation baseline was bubbled with air for >24hr and then stored at the calibration temperature for >24hr before conducting the oxygen calibration. After the MIMS system pressure was stabilized at < 4.0 x 10^−7^ mbar, we pipetted 1.5 mL of air-saturated medium into the sample holder, inserted the standard taper plexiglass closure with the pH sensor forcing all air and some media out of the measurement chamber, then ran the experimental light sequence (see P vs. E section). Once completed, a small amount of Na_2_S_2_O_4_ was added to remove all dissolved oxygen from the medium, and the zero oxygen level was recorded. In the MIMS system, the flux of molecules from the aqueous medium across the membrane into the vacuum space depends on the pressure differential, and therefore we used the oxygen to Argon signal ratio to measure the concentration of dissolved oxygen. The saturation oxygen concentration (O_2sat_) as a function of pressure, salinity and temperature was calculated following Garcia and Gordon [30] (Calculations in S4 Text).

### Statistical analysis

For method validation, an independent t-test was used to compare the carbon uptake rates using the pHOS, and carbon growth over a 5-hour period, both done on the same culture. For detection limit based on the t-score calculation, we divide the relative contribution of a measurement error from an individual sample (x- $\bar{x}$) by the standard error of the mean of the samples (s / √n; Eq 16).

$$t=\frac{x-\bar{x}}{s/\sqrt{n}}$$ (16)

We determined the mean of the t-score for each sample at each Chla concentration (degree of freedom n = 7 light levels– 1 replication factor = 6), and fitted it with a power function. The fitted curve was compared to critical value (CV) from a standard t-table to determine the minimal Chla concentration for effective results. All statistical analysis was conducted using Python 3.5.3 and Scipy 1.8.2 in a Jupyter notebook.

## Results and discussion

### Carbon uptake detection and validation

DIC changes reflect the rate of carbon flux into and out of algal cells, yet these rates might be not the same as carbon uptake and respiration rates, especially for eukaryotic algal cells in which DIC might be concentrated in chloroplasts prior to fixation, resulting in the efflux of DIC from the cell by passive diffusion down the concentration gradient during the post illumination period [31]. Therefore, it is important to confirm the biological mechanisms associated with the measured DIC changes, and in this study we tested and validated the DIC uptake measurement using the pHOS system. CO_2_ released from respiration of cellular organic matter is another important physiological parameter, however due to the lack of methodologies to estimate short term respiration rates, this process could not be quantified independently. The experiment was conducted by establishing the cells in steady-state (continuous light, constant temperature, excess nutrients and mid-log phase exponential growth). Light intensity was 110 ± 10 μmole quanta m^-2^ s^-1^, which is approximately the saturation irradiance for this strain [32]. The samples for testing were concentrated to the optimal concentration ranges ([Chla] >1.5 μg ml^-1^ and OD_750_ < 0.35), and the P vs. E response was measured and fitted with Eq 17 [22].

**Table 2:** P vs. E parameters with data fitted using equations from Platt et al. [22], with sensitivity test results.

	P_max_ (Carbon)	α (Carbon)	β (Carbon)
Baseline	154.79	1.11	0
	±12.97	±0.15	NA
A_T_ + 1%	+0.76%	+0.75%	0
AT—1%	-0.76%	-0.75%	0
pH + 0.01	+1.11%	+1.12%	0
pH—0.01	-1.10%	-1.11%	0

$$P=P_{\max}(1-e^{-\frac{\alpha I}{P_{\max}}})e^{-\frac{\beta I}{P_{\max}}}$$ (17)

**Fig 5:**
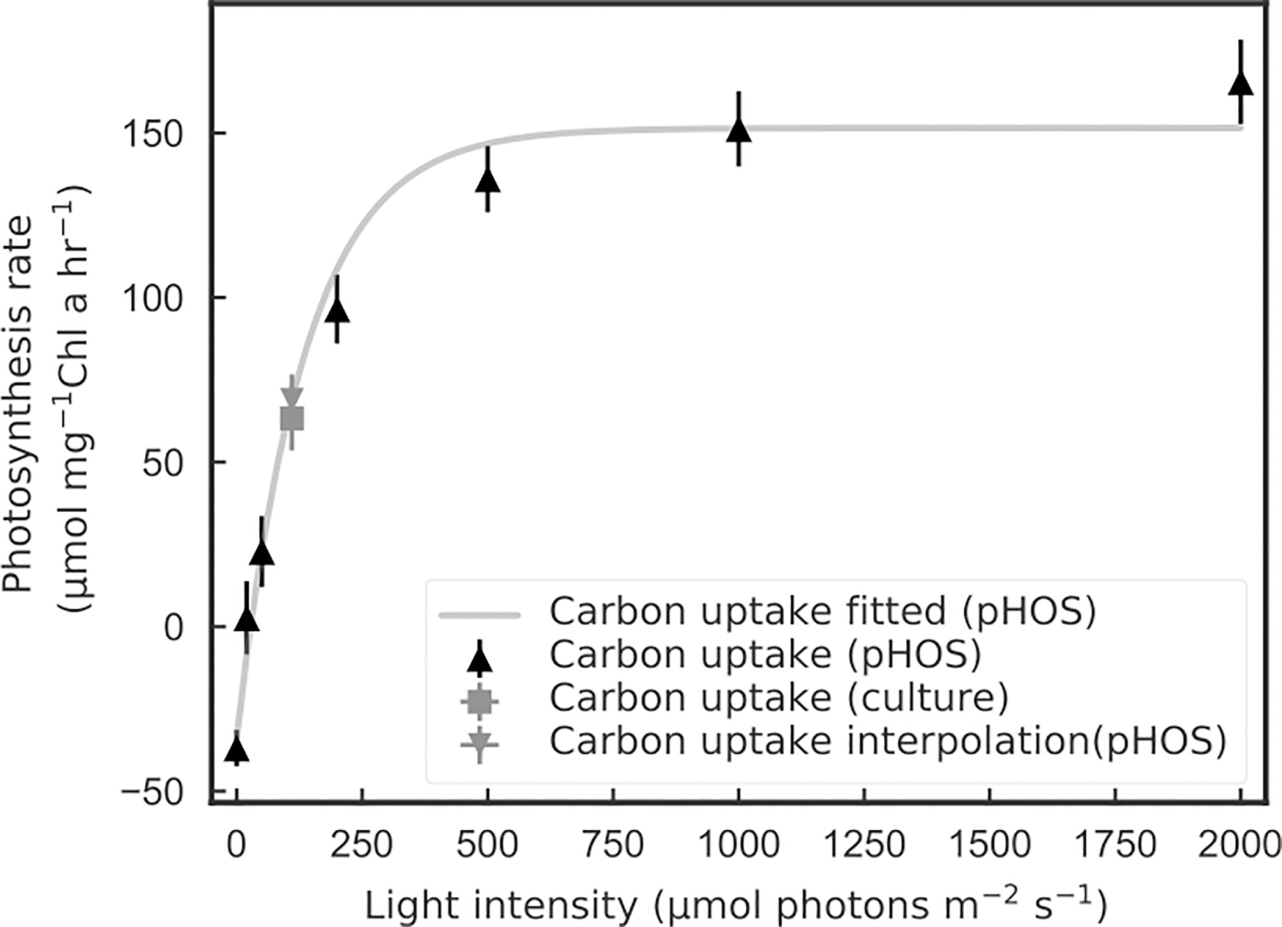
Carbon uptake rates vs. irradiance (n = 3) using pHOS. The estimated net carbon growth rate for a separate culture validation experiment at 110 μmole quanta m^-2^ s^-1^ is indicated by the closed square and the corresponding interpolation from the pHOS data at the same irradiance is shown in the inverted triangle (n = 6). Carbon uptake rates determined from these two approaches are not significantly different from each other (p > 0.05).

P_max_ is the photosynthetic rate at the optimal light condition, α and β (if present) are the parameters that control the initial slope of the curve and the photoinhibition factor, respectively. P vs. E results for carbon are shown in Fig 5 with fitted curves and the replicability of the measurement is shown in Table 1. Considering the potential error in the pH and alkalinity measurements, we performed a sensitivity analysis to determine the uncertainty in our results by manually adding in errors that could have occurred during the measurement. Our titrated A_T_ results showed less than 1% standard deviation, and the pH calibration drift between measurements were less than 0.01 pH units. Both errors together introduce approximately a 1% uncertainty in the final results (Table 2).

Independent from the samples collected for P vs. E measurements, we grew 6 *P*. *tricornutum* cultures under the same culture conditions (excess nutrients, 25°C and 110 μmole quanta m^-2^ s^-1^ continuous light) and determined the natural log (base e) exponential growth rates (μ) at steady state of the cultures by measuring their optical density at 750 nm (OD_750_) over time. During mid-log phase of exponential growth, the OD_750_ was well correlated to cell concentration, POC and Chla. The Chla specific carbon uptake rates (P_Chl_) for these samples were then calculated using Eq 18.

$$P_{Chl}=\mu \frac{\left[POC\right]}{\left[Chla\right]}$$ (18)

The estimate for P_chl_ determined with Eq 9 for the bulk culture, and separately the P_chl_ value determined with the pHOS, interpolated to the same growth irradiance, agreed well (62.5 ± 9.5 and 68.0 ± 7.9 μmole carbon mg^-1^ Chla h^-1^, respectively). The pHOS data, the P vs. E curve fit to the pHOS data, and the two estimates at 110 μmole quanta m^-2^ s^-1^ for the bulk culture and the pHOS interpolation are shown in Fig 5. The agreement in the rate of carbon fixation between the pHOS P vs. E interpolated to 110 μmole quanta m^-2^ s^-1^ and the mid-log phase batch culture provides the primary validation of our pHOS method. Further support for this is provided by Hopkinson et al. [31] who demonstrate that the rate of carbon fixation intracellularly is >10 times slower than the exchange rates between the cell and the medium, thus the 2 min measurement duration for pHOS rates for each light level is more than sufficient to ensure balanced CO_2_ exchange for carbon fixation.

### Quantum yield dynamics during low-to-high light acclimation

**Fig 6:**
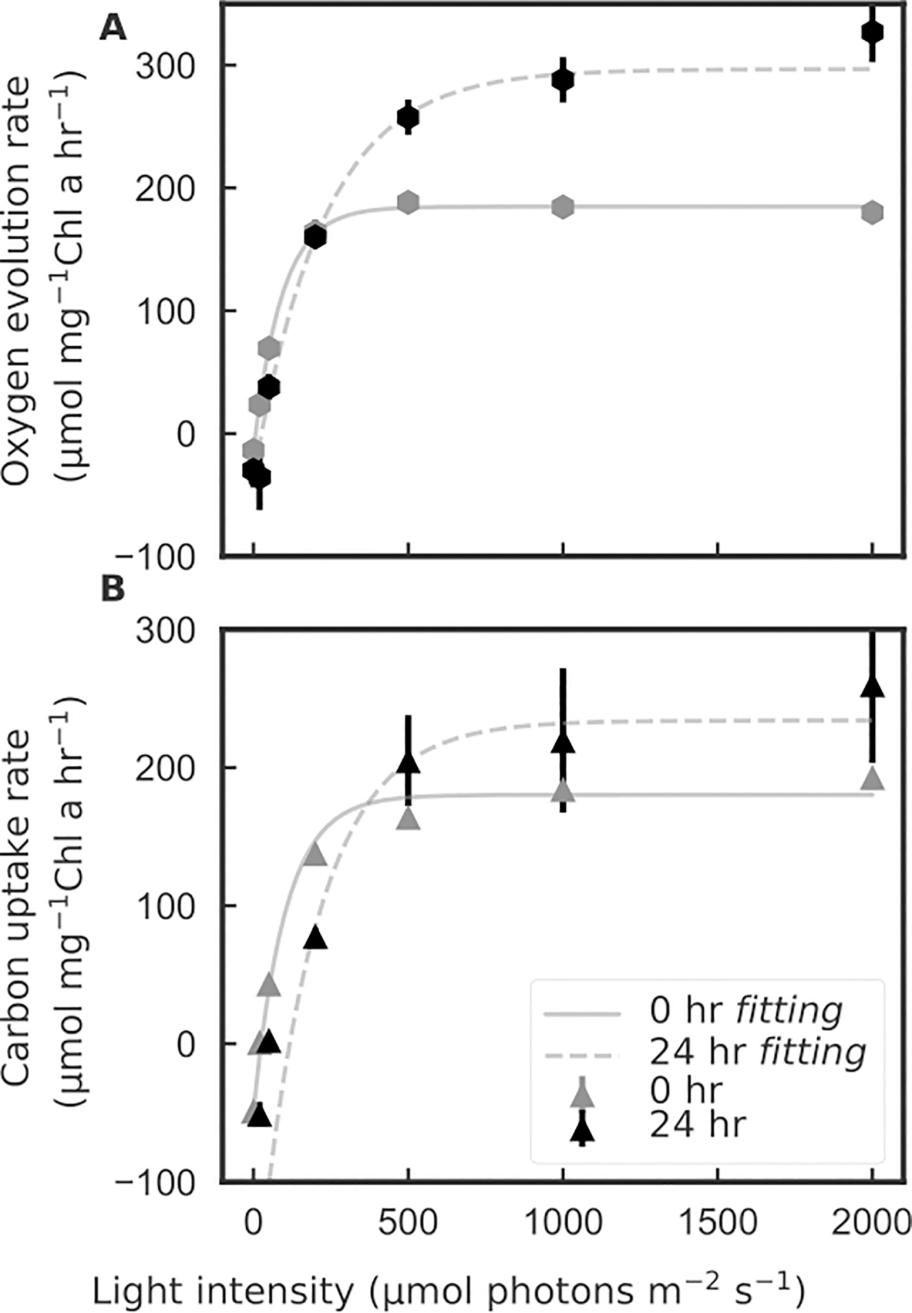
**(A) Chlorophyll a specific oxygen evolution, and (B) carbon uptake rates, as a function of light intensities measured with the pHOS-MIMS system at 0 and 24 at the start and end of the 24 hours low-to-high light acclimation.** Error bars are standard deviations for n = 3; for values with no error bar shown, the standard deviation was smaller than the symbol in the figure. The initial slopes (μmole carbon or oxygen mg^-1^ Chla h^-1^ [μmole quanta m^-2^ s^-1]-1^) of the fitted curves were determined using equations developed by Jassby and Platt [35] with values as follows: 2.17 for oxygen evolution at 0 hour; 1.46 for oxygen evolution at 24 hours; 2.12 for carbon uptake at 0 hour; 2.36 for carbon uptake at hour 24 hours.

To investigate the dynamics of Φ in a non-steady state environment, we shifted low light acclimated *P*. *tricornutum* cultures to super-saturating irradiance (110 to 750 μmole quanta m^-2^ s^-1^), and measured the Φ of oxygen evolution and DIC uptake during the 24 hour high light acclimation. Significant physiological changes in high light treated cells were observed, and the measured shift in cellular Chla concentrations, particulate carbon and nitrogen (POC/PON), and Fv/Fm responses at time 0 and 24 hours (Table 3) are consistent with previous published studies on *P*. *tricornutum* acclimated to different light conditions [33,34], confirming the detected physiological changes are correlated to acclimation to high light stress. From P vs. E results we found similar initial slopes at 0 and 24 hours for both the Chla specific oxygen evolution and carbon uptake, whereas for the high light region > 500 μmole quanta m^-2^ s^-1^ the rates increased at the 24 hour time point (Fig 6).

**Fig 7:**
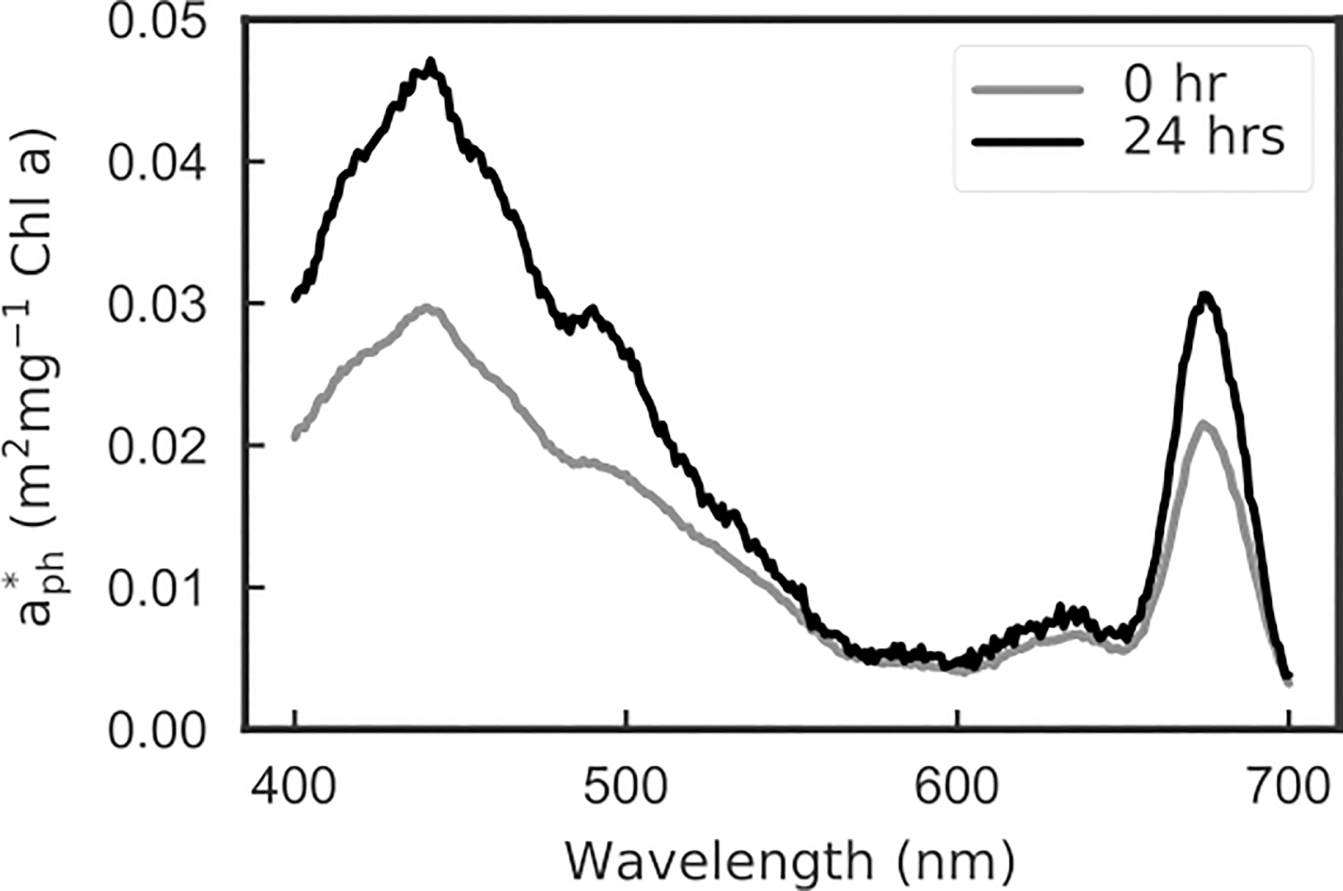
Spectral chlorophyll a specific absorption coefficients measured at 0 and 24 hours during the 24 hours low-to-high light acclimation.

The P vs. E response pattern is consistent with previous studies on a diatom *Skeletonema costatum* [2,36], for P vs. E determined for low light and high light acclimation conditions (50 and 1200 μmole quanta m^-2^ s^-1^, respectively). This effect is likely the combined result of decreases in cellular Chla concentrations and pigment packaging effects, which is inferred by the increased Chla specific absorption coefficient [37,38] (Fig 7).

**Fig 8:**
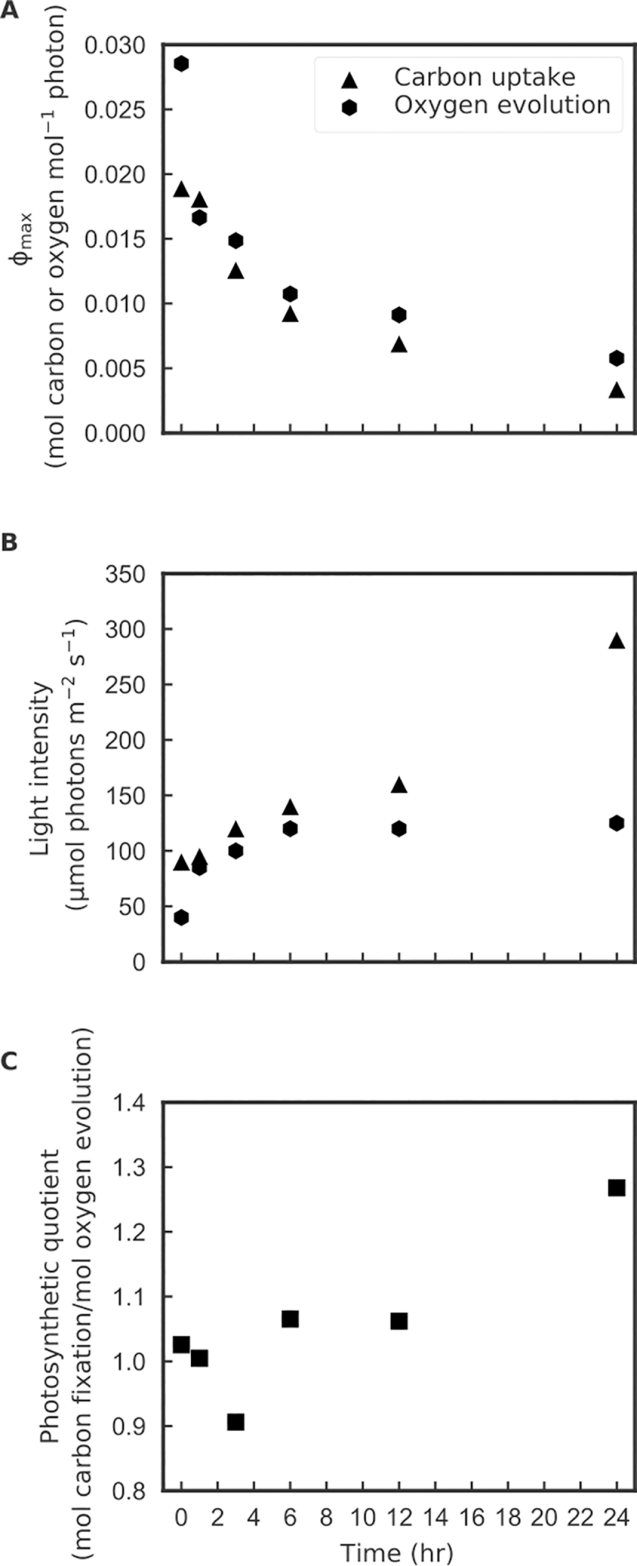
**Observations during the 24 hours low-to-high light acclimation of (A) Maximal observed quantum yield for net photosynthesis, (B) the corresponding light intensities where maximal net quantum yield was observed, (C) Photosynthetic quotient of P**_**max**_. All data points were determined from a P vs. E curve fitted to the mean of 3 replicates; see Fig 6 for typical statistics for the replicates that were fitted.

Note that the calculation of Φ_max_ in our method is determined from the peak value of the Φ vs. E curve, and not from the initial slope of a P vs. E curve, as is common practice [39,40]. At low light, basal respiration can be large relative to photosynthesis and at very low light it can even exceed photosynthesis (S3 Fig). Our method does not calculate Φ_max_ at the very low light levels as might be expected from short term 14-C tracer studies that estimate gross photosynthesis [2], since pHOS is considered to measure net photosynthesis. In our study the observed Φ_max_ dropped significantly between the start and end of the acclimation to high light because both the chla-specific absorption increased ~2x (Fig 7) due to lower pigment packaging effects [41], and also the light intensity corresponding to the Φ_max_ increased significantly during acclimation (Fig 8B). The combination of these factors resulted in a greater than 5x reduction in Φ_max_ by the end of the 24 hour experiment. Dark respiration rates in microalgae have been reported to be positively correlated with growth rates [42,43]. In our study, a higher growth rate would be expected after 24 hours of acclimation to higher light with a concomitant increase in dark respiration rates. The dynamics of Φ in response to light indicate that the acclimation process is non-linear and the changes in carbon uptake and oxygen evolution Φ are not synchronized (S3 Fig). The Φ for oxygen evolution dropped rapidly at low light within the first hour of high light treatment, however the carbon uptake did not change until the 3-hour time point. Also, by 24 hours the carbon:oxygen ratio became lower (higher oxygen evolution than carbon uptake). Overall, carbon uptake seemed to lag in response to the light change, compared to the oxygen evolution response. Such a non-synchronized change in Φ for oxygen evolution and carbon uptake might be related to the transcriptional regulations in *P*. *tricornutum*, reported by Nymark et al. [14] who found that genes that regulate carbon metabolism, respiration and the Calvin-Benson cycle changed later in the 24 hour acclimation phase, while the down regulation of light harvesting antenna genes responded immediately. We designed our experiments to be comparable to Nymark et al. [14] by using the same culturing medium, strain of *P*. *tricornutum*, and very similar culturing and sampling protocols. Our data show the utility of the pHOS-MIMS system for physiological validation of independent molecular level regulation of metabolic processes.

For the high light acclimation time-series, Fig 8A shows changes in Φ_max_ for oxygen and carbon, and changes in the irradiance where the Φ_max_ is attained for the same data. Over the 24 hour acclimation to high light the Φ_max_ for both carbon and oxygen drop and the irradiance at which Φ_max_ was observed shifted higher (Fig 8B). Furthermore the PQ for the fitted P_max_ for both carbon and oxygen showed an initial drop at the 3 hour time point followed by a steady increase, which is consistent with our previous inference that oxygen shifts start sooner than carbon shifts (Fig 8C). Photorespiration might be important in the shift to super-saturating light, reducing the oxygen evolved and hence leading to a lower PQ during the first few hours (Fig 8C). The increase of PQ in the later acclimation period might have resulted from stronger alternative electron transport [44,45]. The overall trend of a strong decrease in Φ_max_ for both oxygen and carbon following the shift to super-saturating light is likely correlated to the reduction of cellular light harvesting pigments, rebalancing of PSII to PSI, and reaction center damage that has not been repaired [46,47]; These physiological changes are also reflected as decreases in Fv/Fm (Table 3). As these shifts occurred there were also strong changes over 24 hours in cellular properties, as shown by observed decreases in Chla cell^-1^, and increases in C:Chla and the chlorophyll specific absorption coefficient (a*_ph_). These cellular property changes are consistent with a physiological transition from low light to high light acclimation.

### System operation and implementation

We found the integration of our pHOS and the MIMS to the ALG instruments bioreactor system provided robust data. Although the pHOS system has some novel circuitry and software and we optimized the membrane for the MIMS, all of these novel elements and improvements are well documented here and in the SI. Therefore, based on our current system design, hardware, software and integration, and documentation in the manuscript and the SI, the system could be easily replicated. The pHOS system to estimate carbon uptake from an aqueous medium can be run independent of oxygen measurements. All of the electronic parts required for the pHOS system can be sourced on-line using the information provided in the methods section, and the software for operation is provided in the SI. We used the MIMS for oxygen in part because we intend to use it for other gases in the future but the pHOS system can also be easily used with other oxygen measuring systems such as a Clark electrode or an optode.

**Table 3:** Physiological parameters measured at 0 and 24 hours during the low-to-high light acclimation (n = 3 for all samples).

Time	C (pg cell^-1^)	N (pg cell^-1^)	Chla (pg cell^-1^)	C:N	C:Chl	Fv/Fm
0 hour	11.44 ± 1.45	1.77 ± 0.07	0.58 ± 0.05	6.47	21.07	0.71±0.007
24 hours	15.46 ± 2.10	2.08 ± 0.21	0.21 ± 0.02	7.43	77.32	0.59±0.005

## Conclusions

Photoacclimation related NPQ response and transcriptional regulation can affect the fate of electrons in the photosystems, resulting in changes in Φ and its response to light at various time-scales from short-term perturbations to steady-state acclimation. Our method successfully detected the change of instantaneous Φ in relation to irradiance variation from 0–2000 μmole quanta m^-2^s^-1^, allowing simultaneous tracking of carbon uptake and oxygen evolution quantum efficiencies during acclimation, in addition to the classic chlorophyll variable fluorescence measurements. We found the optimal concentration for measurement is between [Chl a] >1.5 μg ml^-1^ and OD_750_ < 0.35, which allows sufficient signal for sensor detection and minimizes light attenuation within the bioreactor by cellular absorption. The system configuration as described is ideal for laboratory algal physiological experiment, yet the modular components can be integrated with other instrument for different purposes.

Our results demonstrate that there is a non-synchronous response of carbon uptake and oxygen evolution during the acclimation period from low to high light that is consistent with transcriptomic data [14]. These physiological observations could provide quantitative data for industrial applications such as production simulation in dynamic light environments, and energy utilization per unit of carbon fixed for life cycle analysis. They also inspired testable hypotheses regarding photosynthetic electron transport efficiency related to cellular mechanisms that could be pursued with the pHOS-MIMS system combined with other measurements, for example transcriptomics, variable chlorophyll fluorescence, mutant strains and stable oxygen isotopes. The novel pHOS system described here allows for the resolution of carbon dynamics with temporal resolution sufficient for diel studies and for resolving acclimation to environmental changes on time-scales of hours to days, and was validated with classical observations of rates of carbon growth in microalgae cultures. The integration of the pHOS with a MIMS system demonstrates an ability to resolve both carbon and oxygen dynamics during light and dark periods that were used to construct P vs. E curves from limiting to super-saturating light within 30 minutes. This type of simultaneous oxygen and carbon data is essential for metabolic modeling of mass fluxes and contributes to the integration between classic photo-physiology and the rapidly emerging interest in computational biology. Furthermore, the ability to resolve physiological responses to changing light conditions at time scales of minutes is highly relevant for understanding growth rates of microalgae within the near surface turbulent mixing layer of aquatic ecosystems or commercial production systems.

## Supporting information

S1 Fig **Examples of pH and CO**_**2**_ **signals measured with pHOS (A) and MIMS (B).** The gray bars show the spans and intensities of the light periods. (A) Using pHOS, the change of pH at the different light steps mostly resulted from HCO_3_^-^ uptake and CO_3_^2-^ dehydration, therefore pH was less affected by the limiting HCO_3_^-^ dehydration step. (B) Using MIMS the rate of CO_2_ consumption during the light exposure decreased over time, presumably due to increases in the rate of HCO_3_^-^ dehydration. During the dark periods, the CO_2_ concentration first increased rapidly, and then slowed down as CO_2_ hydration rate increased.
(TIFF)

S2 Fig Simultaneous measurement of oxygen evolution (A) and carbon uptake (B) for 5 samples with different Chla concentrations.
(TIFF)

S3 Fig Dynamic changes of carbon uptake and oxygen evolution quantum yield as a function of instantaneous irradiance during the 24 hours high light acclimation. (TIFF)

S1 Text Measuring dissolved CO2 vs. measuring pH. (DOCX)

S2 Text Parameters for A_C_ calculation. (DOCX)

S3 Text Nernst equation validation. (DOCX)

S4 Text Equations for computing saturation oxygen concentration. (DOCX)
