# Supplementary figures and images for: Simultaneous quantum yield measurements of carbon uptake and oxygen evolution in microalgal cultures
Source: PLoS One. 2018 Jun 19;13(6):e0199125. doi: 10.1371/journal.pone.0199125 (PMC6008153; doi:10.1371/journal.pone.0199125)

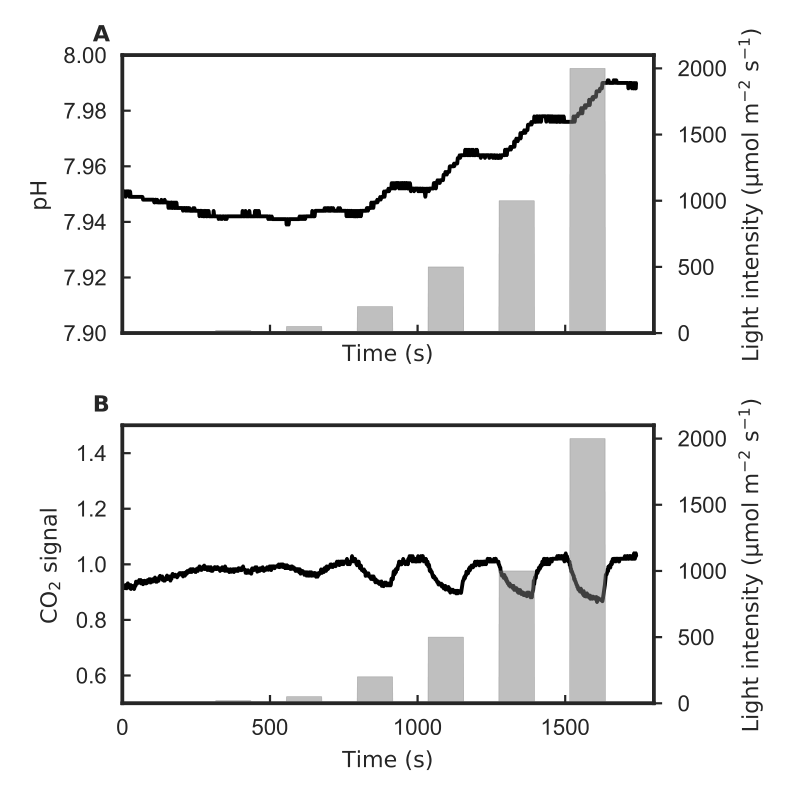

Supplement: S1 Fig — Examples of pH and CO2 signals measured with pHOS (A) and MIMS (B). The gray bars show the spans and intensities of the light periods. (A) Using pHOS, the change of pH at the different light steps mostly resulted from HCO3- uptake and CO32- dehydration, therefore pH was less affected by the limiting HCO3- dehydration step. (B) Using MIMS the rate of CO2 consumption during the light exposure decreased over time, presumably due to increases in the rate of HCO3- dehydration. During the dark periods, the CO2 concentration first increased rapidly, and then slowed down as CO2 hydration rate increased. (TIFF) [file pone.0199125.s001.tiff]

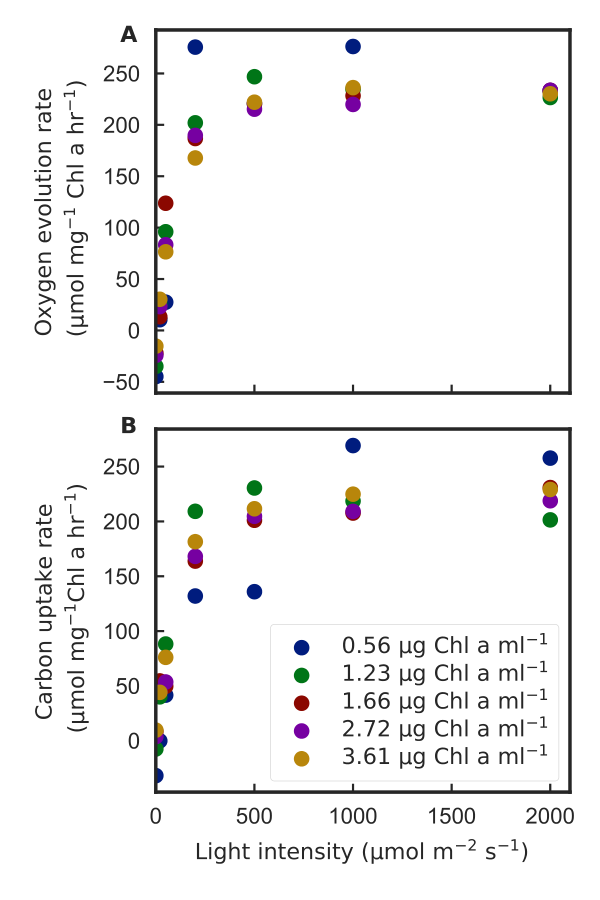

Supplement: S2 Fig — Simultaneous measurement of oxygen evolution (A) and carbon uptake (B) for 5 samples with different Chla concentrations. (TIFF) [file pone.0199125.s002.tiff]

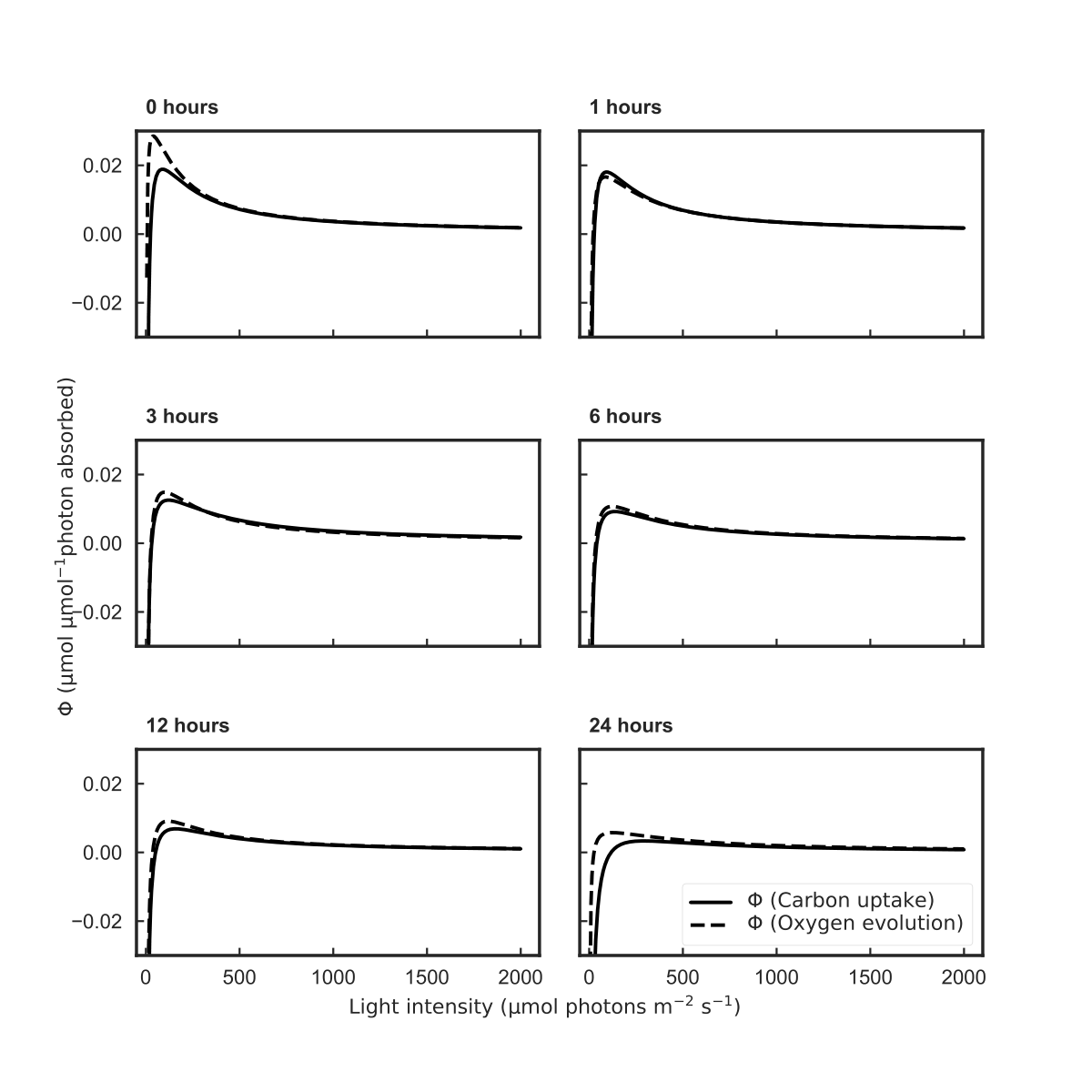

Supplement: S3 Fig — (TIFF) [file pone.0199125.s003.tiff]
