# Supplementary material for: Simultaneous quantum yield measurements of carbon uptake and oxygen evolution in microalgal cultures
Source: PLoS One. 2018 Jun 19;13(6):e0199125. doi: 10.1371/journal.pone.0199125 (PMC6008153; doi:10.1371/journal.pone.0199125)
Supplement: S1 Text — (DOCX) [file pone.0199125.s004.docx]

**Measuring dissolved CO2 vs. measuring pH**

There are three fundamental approaches for determining carbon uptake rates in microalgae: (1) the accumulation of particulate organic carbon (POC) in algal cells, (2) the removal of dissolved inorganic carbon (DIC) from the culture medium, and (3) the use of ^14^C or ^13^C bicarbonate added as a tracer to the medium. The extra step of separating the POC out of a liquid sample (a critical step for POC measurement) can introduce error, add time to each measurement, and requires relatively large algae biomass. On the other hand the isotope tracer methods generally require a relatively long incubation (30-60 min) to achieve reasonable results. Therefore, a method using direct measurement of DIC change in the medium would be faster and be a valuable option for dynamic carbon uptake measurements. In terrestrial plant physiology the common approach for directly estimating carbon uptake is accomplished by determining the change in CO_2_ concentration in a closed space containing the live plant (Oja et al., 2007). However, applying this methodology to microalgae is not straightforward as the algae are immersed in fluid. The CO_2_ in the headspace over a liquid culture could be measured but the slow gas exchange between air and the liquid phase reduces measurement sensitivity. An example can be found in the experiments conducted by Oakley et al. (2012), in which the measured CO_2_ concentration in the headspace continued to decrease after the illumination source was switched off. For a direct CO_2_ estimate in solution using a MIMS (Hopkinson et al. 2011), the sluggish conversion between HCO_3_^-^ to CO_2_ (Reinfelder, 2011) can mask the biological DIC change. For example, if the rate of CO_2_ uptake exceeds the rate of HCO_3_^-^ dehydration, only the latter rate can be detected by measuring CO_2_ using a MIMS.

During our carbon uptake measurements using the pHOS system, a series of alternating light and dark periods were applied during the 30 min measurement cycle. Over this 30 min cycle, we used 6 discrete light intensities from 20 - 2000 µmole Quanta m^-2^sec^-1^ oscillating the light on and off for 2 minutes each. We determined the oscillating pH signal due to cellular switching between photosynthesis and respiration in the alternating light/dark regime. The pH signal shift tracked the shifts from light to dark periods very well, and the pH resolution was sufficient for reliable calculations of carbon uptake rates.

Although we could simultaneously measure CO_2_ and O_2_ changes with the MIMS, we did not use MIMS CO_2_ uptake estimates for the following reasons. The net formation of CO_2_ from the dehydration of HCO_3_^-^ , according to (Schulz et al., 2006), can be described as: (1)

where k_+_ and k_-_ are the rate constants for HCO_3_^-^ dehydration and CO_2_ hydration. Assuming pH = 8 and A_C_= 2000 μmol/kg in a standard seawater medium, the starting concentrations of HCO_3_^-^ and CO_2_ was calculated to be 1779.4 μmole kg^-1^ and 12.5 μmole kg^-1^, respectively (CO2Calc, Robbins et al. 2010). At equilibrium, the CO_2_ hydration rate is equal to the HCO_3_^-^ dehydration rate, and k+ can be estimated to be 3.64 × 10^-4^ s^-1^ (Schulz *et al.*, 2006), and therefore, by calculation, the bi-directional flux between HCO_3_^-^ and CO_2_ is equal to 0.65 μmole kg^-1^ s^-1^, and k_-_ is equal to 5.18 × 10^-2^ s^-1^. After the activation of photosynthesis, CO_2_ is depleted faster than HCO_3_^-^, e.g. at rates of 1.9 × 10^-11^ μmole cell^-1^ s^-1^ and 0.9 × 10^-11^ μmole cell^-1^ s^-1^ in *P. tricornutum* (Hopkinson *et al.*, 2011). Considering the cell density at 5 × 10^9^ cells kg^-1^ (within the range of our recommended density), the rates of CO_2_ and HCO_3_^-^ uptake are 9.5 × 10^-2^ μmole kg^-1^ s^-1^ and 4.5 × 10^-2^ μmole kg^-1^ s^-1^. As the equilibrium between CO_2_ and HCO_3_^-^  shifts due to carbon uptake, a new CO_2_ formation rate can be calculated as: (2)

 increases at a rate of 4.91 × 10^-3^ μmole kg^-1^ s^-2^ after the activation of photosynthesis, in other word, at a given condition based on the above assumptions, it would take approximately 20 s before the rate of CO_2_ formation from dehydration of HCO_3_^-^ would equal the CO_2_ uptake rate, however this is not actually in steady-state because the carbonate system speciation is changing. The specific time to reach this “steady state” depends on the medium chemistry and CO_2_ uptake rates.
