# Supplementary material for: Simultaneous quantum yield measurements of carbon uptake and oxygen evolution in microalgal cultures
Source: PLoS One. 2018 Jun 19;13(6):e0199125. doi: 10.1371/journal.pone.0199125 (PMC6008153; doi:10.1371/journal.pone.0199125)
Supplement: S2 Text — (DOCX) [file pone.0199125.s005.docx]

**Parameters for AC calculation**

In this study, artificial seawater media (ASW; http://www3.botany.ubc.ca/cccm/ NEPCC/esaw.html) was used for cultivation and also for the pH measurements. The salinity of the ASW was adjusted to 36 practical salinity units (PSU), and therefore, we adopted the ionization functions K_1_ and K_2_ that are optimized for salinities in the range 20 to 40 units (Dickson & Millero, 1987). Values of pK_1_ (Equation 3) and pK_2_ (Equation 4) are functions of temperature (T) and salinity (S).

 (3)

 (4)

As described in the main article, the ASW had nutrients and other components required for cell growth and some of these can contribute to A_T_. In this more complex culturing medium, the A_T_ remained stable because the CO_2_ was depleted due to algal assimilation of CO_2_ and efflux of OH^-^, as previously described in detail in Wolf-Gladrow et al (2007).

Equations for boron and phosphate contributed to the alkalinity calculation as follows:

 (5)

 (6)

 (7)

 (8)

 (9)

 (10)
