# Supplementary material for: Simultaneous quantum yield measurements of carbon uptake and oxygen evolution in microalgal cultures
Source: PLoS One. 2018 Jun 19;13(6):e0199125. doi: 10.1371/journal.pone.0199125 (PMC6008153; doi:10.1371/journal.pone.0199125)
Supplement: S3 Text — (DOCX) [file pone.0199125.s006.docx]

**Parameters for AC calculation**

In this study, artificial seawater media (ASW; http://www3.botany.ubc.ca/cccm/ NEPCC/esaw.html) was used for cultivation and also for the pH measurements. The salinity of the ASW was adjusted to 36 practical salinity units (PSU), and therefore, we adopted the ionization functions K_1_ and K_2_ that are optimized for salinities in the range 20 to 40 units (Dickson & Millero, 1987). Values of pK_1_ (Equation 3) and pK_2_ (Equation 4) are functions of temperature (T) and salinity (S).

 (3)

 (4)

As described in the main article, the ASW had nutrients and other components required for cell growth and some of these can contribute to A_T_. In this more complex culturing medium, the A_T_ remained stable because the CO_2_ was depleted due to algal assimilation of CO_2_ and efflux of OH^-^, as previously described in detail in Wolf-Gladrow et al (2007).

Equations for boron and phosphate contributed to the alkalinity calculation as follows:

 (5)

 (6)

 (7)

 (8)

 (9)

 (10)

 (11)

**Nernst equation validation**

pH electrodes from different manufacturers do not always produce consistent, reproducible measurements and are not of consistent quality. Therefore for high precision measurements of small pH changes, it is essential to calibrate each pH electrode’s performance before starting experiments. We validated the performance of the Cole-Parmer glass liquid-filled pH electrode using standard NBS buffers at temperatures ranging from 20 to 30 °C (with 1 °C increment resolution), and then compared corresponding electrode response to the theoretical Nernst response under ideal conditions (Table S1).

The electric potential from the digital reading was then calculated using Equation 12.

 (12)

The pH of the three NBS buffers were calculated based on temperature calibrations (Hetzer et al. 1977; Ashton & Geary 2005). The electrode response at each temperature was then calculated by taking the slope of the electric potential vs. pH, and the Nernst response was calculated using Equations 13-14.

 (13)

 (14)

The selected Cole-Parmer liquid-filled glass pH electrode produced consistent response efficiencies of 96% within the range of temperatures tested (S1 Table). These consistent efficiencies suggested the electrode used was in good condition and the pH calibration minimized any bias in measurement efficiency by offsetting the differences.

| S1 Table. pH electrode voltage vs. Nernst equation validation parameters | | | | | | |
| --- | --- | --- | --- | --- | --- | --- |
| **T (°C)** | **pH 4.01** | **pH 7** | **pH 10.01** | **s_Nernst_**  **(mV/pH)** | **s_electrode_**  **(mV/pH)** | **% slope** |
| 20 | 4.0015 | 7.0223 | 10.0640 | 58.10 | 55.88 | 96.17% |
| 21 | 4.0022 | 7.0185 | 10.0534 | 58.30 | 56.07 | 96.18% |
| 22 | 4.0030 | 7.0149 | 10.0429 | 58.50 | 56.26 | 96.17% |
| 23 | 4.0038 | 7.0113 | 10.0327 | 58.70 | 56.46 | 96.19% |
| 24 | 4.0048 | 7.0079 | 10.0226 | 58.89 | 56.65 | 96.19% |
| 25 | 4.0059 | 7.0046 | 10.0128 | 59.09 | 56.84 | 96.19% |
| 26 | 4.0070 | 7.0014 | 10.0031 | 59.29 | 57.03 | 96.18% |
| 27 | 4.0082 | 6.9983 | 9.9937 | 59.49 | 57.20 | 96.15% |
| 28 | 4.0095 | 6.9953 | 9.9845 | 59.69 | 57.38 | 96.14% |
| 29 | 4.0109 | 6.9925 | 9.9754 | 59.89 | 57.57 | 96.13% |
| 30 | 4.0124 | 6.9897 | 9.9666 | 60.08 | 57.76 | 96.13% |
