# Supplementary material for: Simultaneous quantum yield measurements of carbon uptake and oxygen evolution in microalgal cultures
Source: PLoS One. 2018 Jun 19;13(6):e0199125. doi: 10.1371/journal.pone.0199125 (PMC6008153; doi:10.1371/journal.pone.0199125)
Supplement: S4 Text — (DOCX) [file pone.0199125.s007.docx]

**Equations for computing saturation oxygen concentration**

 (15) Equation S16

 (16)

* Oxygen density at 1.43905 mg/ml, and molecular weight at 32g mol^-1^

At salinity 36 and temperature at 25°C, saturation oxygen level equals 211.85 μmole L^-1^.
